# Comprehensive analysis of the value of RAB family genes in prognosis of breast invasive carcinoma

**DOI:** 10.1042/BSR20201103

**Published:** 2020-05-29

**Authors:** Shitong Lin, Canhui Cao, Yifan Meng, Ping Wu, Peipei Gao, Wenhua Zhi, Ting Peng, Peng Wu, Lingli Gui

**Affiliations:** 1 Cancer Biology Research Center (Key Laboratory of The Ministry of Education), Tongji Hospital, Tongji Medical College, Huazhong University of Science and Technology, Wuhan, Hubei, China; 2 Department of Gynecologic Oncology, Tongji Hospital, Tongji Medical College, Huazhong University of Science and Technology, Wuhan, Hubei, China; 3 Department of Anesthesiology and Pain Medicine, Tongji Hospital, Tongji Medical College, Huazhong University of Science and Technology, Wuhan, China

**Keywords:** Biologic biomarkers, Breast invasive carcinoma, RAB family genes

## Abstract

**Purpose:** Several RAB family genes have been studied extensively and proven to play pivotal roles in the occurrence and development of certain cancers. Here, we explored commonly expressed RAB family genes in humans and their prognostic significance using bioinformatics, and then identified potential biomarkers of breast invasive carcinoma (BRCA). **Materials and methods:** The prognostic values (overall survival) of RAB family genes in BRCA were obtained using Gene Expression Profiling Interactive Analysis (GEPIA). The expression patterns of RAB family genes and their relationships with clinicopathological parameters in BRCA were measured using the ONCOMINE and UALCAN databases, respectively. Genetic mutations and survival analysis were investigated using the cBio Cancer Genomics Portal (c-BioPortal). Interacting genes of potential biomarkers were identified using STRING, and functional enrichment analyses were performed using FunRich v3.1.3. **Results:** In total, 64 RAB genes were identified and analyzed in our study. Results showed that *RAB1B, RAB2A*, and *RAB18* were up-regulated and significantly associated with poor overall survival in BRCA. Furthermore, their higher expression was positively correlated with clinicopathological parameters (e.g. cancer stage and nodal metastasis status). DNA copy number amplifications and mRNA up-regulation were the main genetic mutations, and the altered group showed significantly poorer overall survival compared with the unaltered group. Functional enrichment analysis of *RAB1B, RAB2A*, and *RAB18* indicated they were closely involved in GTPase activity. **Conclusions:** *RAB1B, RAB2A*, and *RAB18* were up-regulated and significantly correlated with poor prognosis in BRCA. Thus, they could be applied as novel biomarkers of BRCA in future studies.

## Introduction

Breast invasive cancer (BRCA) is the most common malignancy in females and seriously threatens physical and mental health [1]. It is currently estimated that there will be 276 ,480 new cases and 42 ,170 deaths from BRCA in the United States by 2020 [2]. At present, BRCA treatment mainly includes surgery, radiotherapy, chemotherapy, and endocrine therapy [3]. However, BRCA prognosis remains highly unsatisfactory due to a lack of effective treatments to control the recurrence of distant metastases, especially for advanced patients. In recent years, bio-targeted therapy has been shown to be effective in a variety of cancers, significantly improving prognosis. Therefore, the identification of novel biomarkers is essential for specific treatments in patients.

Rab proteins, which belong to the Ras superfamily, are a class of small molecule GTPases with a molecular mass of about 20–30 ku. They are expressed in membrane-related organelles and vesicles (e.g. endosomes, nuclei, mitochondria, and Golgi apparatus) in various eukaryotic organisms such as yeast, fruit flies, mice, and humans [4]. To date, scientists have identified more than 60 Rab proteins in humans, which have been shown to play regulatory roles in vesicle transport, protein transport, membrane localization, and fusion [5–7]. In addition to their roles in development and physiology, Rab proteins are also closely related to the occurrence and development of cancer [8].

Mutations of Rab proteins alter the efficiency of their interactions with effector molecules, thereby dysregulating the material transport network and causing tumors. Rab proteins are encoded by RAB family genes, and differentially expressed RAB genes have been found in multiple cancers. Studies have shown RAB25 mRNA levels to be significantly up-regulated in ovarian cancer compared with normal tissue, resulting in shorter overall survival (OS) and disease-free survival (DFS) [9,10]. Scientists have also found RAB38 mRNA to be differentially expressed in glioma, and significantly correlated with patient grade progression and poor prognosis [11]. Furthermore, patients with Rab27A (+) or Rab27B (+) exhibit significantly decreased OS compared with those showing Rab27A (−) or Rab27B (−) in hepatocellular carcinoma [12]. However, few studies on RAB family genes have been conducted in BRCA, and little is known about their relevant mechanisms.

Here, we aimed to comprehensively explore the expression, prognosis, and mutations of RAB family genes in BRCA, and identify potential novel biomarkers that may play vital roles in the genesis and development of BRCA.

## Materials and methods

### GEPIA database

GEPIA (http://gepia.cancer-pku.cn/detail.php) is a multi-functional online database, which provides key interactive analysis and customization functions, including tumor or normal differential expression profile analysis, profiling, pathological staging, patient survival analysis, similar gene detection analysis and dimensionality reduction analysi**s** [13]. In our study, we used GEPIA to analyze the prognosis (OS) of RAB family genes in BRCA, and then screened out those genes with statistical significance. The group thresholds were as follows: the group cut-off was ‘Median’, the ‘cutoff-high’ and ‘cutoff-low’ were 50%, axis units were ‘Months’, and *P* value < 0.05 was considered statistically significant

### ONCOMINE analysis

ONCOMINE (https://www.oncomine.org/resource/login.html#) is an online database that contains a variety of cancer microarrays from multiple sources [14]. We used ONCOMINE to crudely measure the mRNA expression levels of RAB family genes which significantly predicted prognosis compared with normal tissue in BRCA. The selection criteria were as follows: fold change = 2, *P*-value < 0.05, and top gene rank was 10%.

### UALCAN analysis

UALCAN (http://ualcan.path.uab.edu/analysis.html) is an effective online database for online analysis and mining of cancer data. Based on the cancer data of (TCGA) database, it can be used for biomarker identification, expression profiling analysis, survival analysis etc [15]. In the present study, we first used it to further measure the expression levels of related genes in BRCA from TCGA samples. Meanwhile, we explored the relationship between mRNA expression levels of potential biomarkers and the clinicopathological parameters of BRCA patients. *P* value < 0.05 was considered statistically significant.

### The Human Protein Altas analysis

The Human Protein Altas (HPA) (https://www.proteinatlas.org/) provides information on the tissue and cell distribution of all 24,000 human proteins. It uses special antibodies and immunohistochemistry technology to check the distribution and expression of each protein in 48 kinds of normal human tissues, 20 kinds of tumor tissues, 47 cell lines and 12 kinds of blood cells [16]. We used this database to further explore protein expression and functions of selected RAB genes in BRCA.

### c-BioPortal

c-BioPortal (https://www.cbioportal.org/) integrates and simplifies the contents of many cancer genomic databases including TCGA, ICGC and GEO, and can analyze somatic mutation spectrum, copy number change, mRNA or miRNA expression change etc [17]. We used it to explore the genetic mutations and their association with BRCA prognosis of potential biomarkers. The selected study was ‘Breast Invasive Carcinoma (TCGA, Firehose Legacy)’ which contained 1108 samples. The select genomic profiles were as follows: ‘Mutations’; ‘Putative copy-number alterations from GISTI’; ‘mRNA Expression *z*-Scores (RNA Seq V2 RSEM)’, and *z*-score threshold was ± 2. The data of ‘OncoPrint’, ‘Cancer Types Summary’ and ‘Survival’ were downloaded after c-BioPortal finished analyzing.

### Functional enrichment analysis

Those genes that interacted with potential RAB family genes were identified using the online search tool STRING (https://string-db.org/) [18]. We then used it to construct the protein–protein interacting network (PPI). Funrich (version 3.1.3) was used to perform functional enrichment analysis according to its operation tutorial [19].

## Results

### Prognostic values (overall survival) of 64 RAB family genes

**Figure 1:**
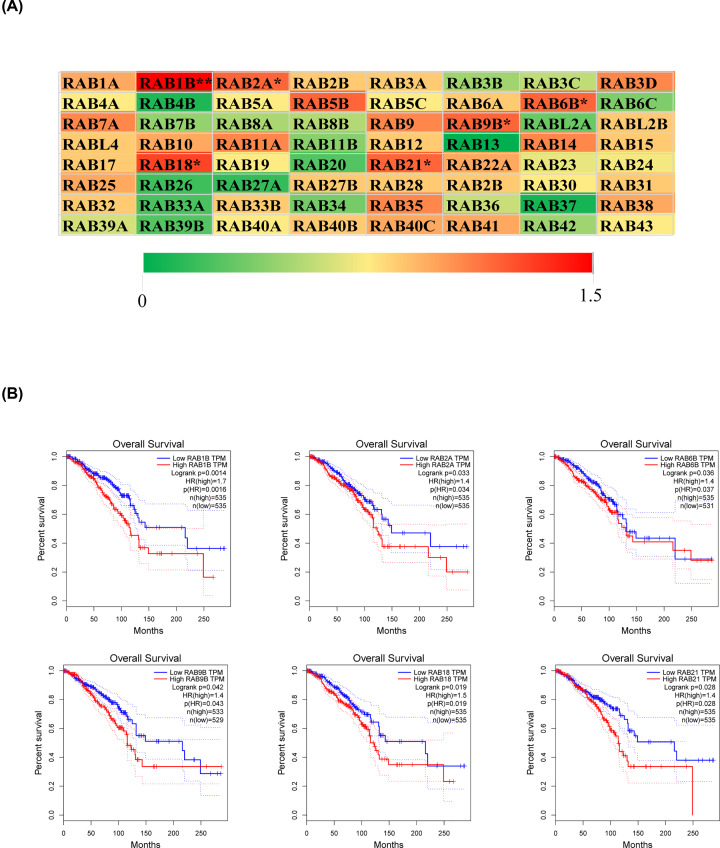
Relationship between RAB family genes mRNA expression levels and overall survival of BRCA (**A**) The prognostic value (OS) of RAB family genes in breast invasive carcinoma using GEPIA. *P<0.05; **P<0.01; ***P<0.001; ****P<0.0001. (**B**) Prognostic value (OS) of *RAB1B, RAB2A, RAB6B, RAB9B*, *RAB18*, and *RAB21* in breast invasive carcinoma.

A total of 64 RAB family genes were identified and analyzed for prognosis (i.e. OS) of BRCA using Gene Expression Profiling Interactive Analysis (GEPIA). As shown in Figure 1A,B, *RAB1B* (HR = 1.7, *P*=0.0016), *RAB2A* (HR = 1.4, *P*=0.034), *RAB6B* (HR = 1.4, *P*=0.037), *RAB9B* (HR = 1.4, *P*=0.043), *RAB18* (HR = 1.5, *P*=0.019), and *RAB21* (HR = 1.4, *P*=0.028) were significantly correlated with poor OS in BRCA. Thus, we considered that these six RAB family genes were the most likely to play vital roles in the occurrence and development of BRCA. Therefore, we selected these six genes for further analysis.

### mRNA transcription levels of six potential biomarkers of BRCA among RAB family genes

**Figure 2:**
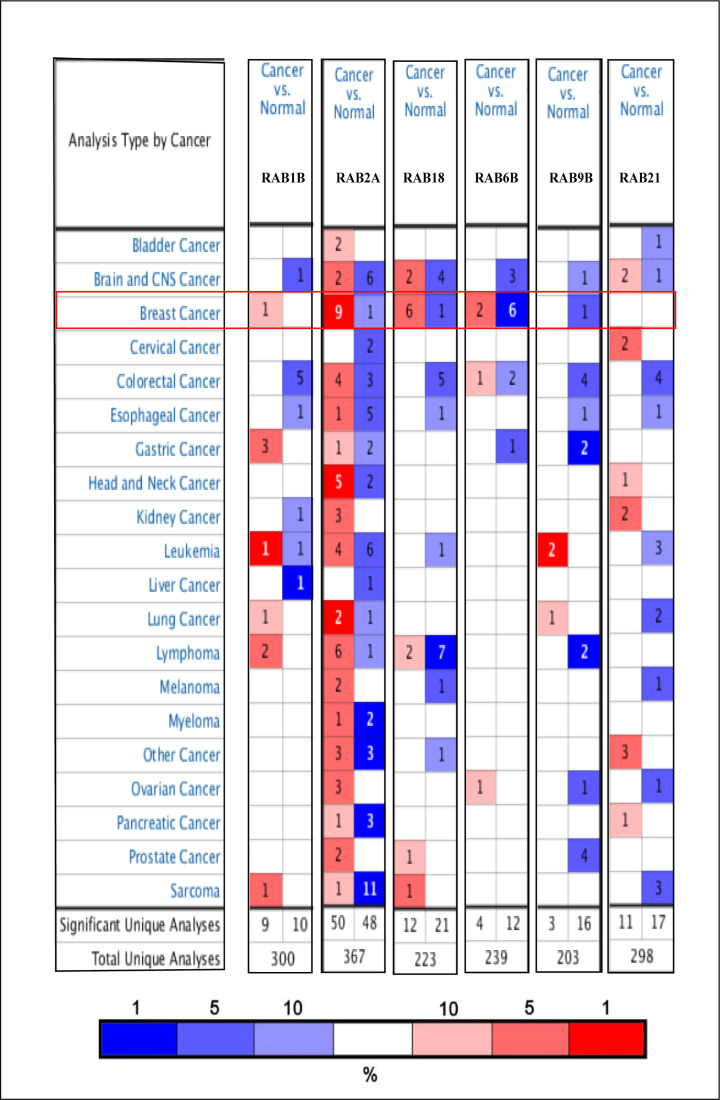
mRNA transcription levels of six potential biomarkers of BRCA among RAB family genes using the Oncomine The number in each cell indicates the number of studies which meets the screening criteria of target genes. The depth of the color is determined by the gene rank percentile below. Red represents over-expression and blue represents under-expression. The selection criteria were as follows: *P*-value was 0.05; gene rank percentile was 10% and fold change was 1.5.

**Figure 3:**
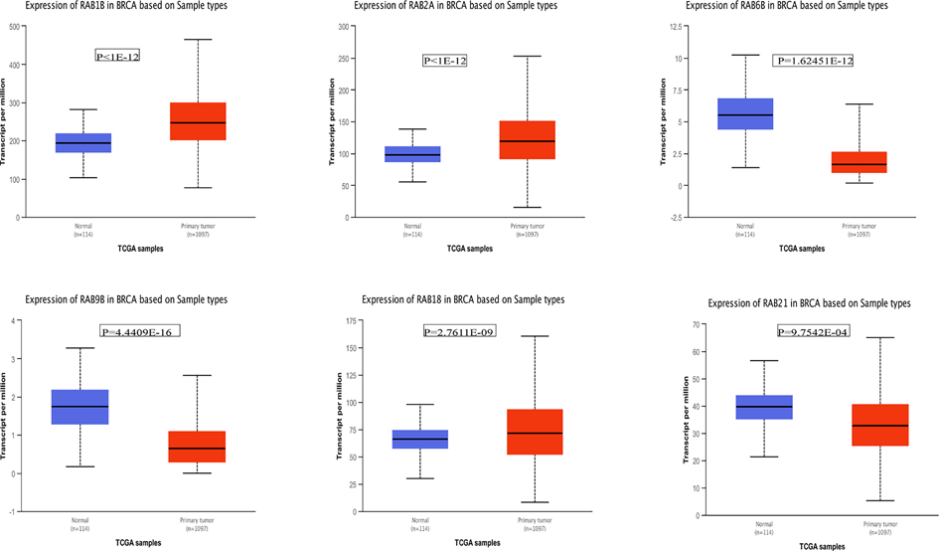
mRNA expression levels of six potential biomarker of BRCA in TCGA samples using UALCAN mRNA expression levels of *RAB1B, RAB2A RAB6B, RAB9B, RAB18*, and *RAB21* based on TCGA samples that consists of 114 normal samples and 1097 primary tumor samples.

We used ONCOMINE and UALCAN to explore the expression levels of potential biomarkers of in BRCA tumor tissue compared with normal tissue. As shown in Figure 2, we found 300, 367, 223, 239, 203, and 298 studies containing *RAB1B, RAB2A, RAB6B, RAB9B, RAB18*, and *RAB21*, respectively. According to figure annotation, *RAB1B, RAB2A, RAB6B, RAB9B*, and *RAB18* were significantly up-regulated in BRCA tissue compared with normal tissue. We then used UALCAN to measure mRNA transcription levels of potential biomarkers of BRCA. As shown in Figure 3, *RAB1B* (*P*<1E-12), *RAB2A* (*P*<1E-12), and *RAB18* (*P*=2.7611E-09) were significantly up-regulated in primary tumor tissue (*n*=1 097) compared with that in normal tissue (*n*=114). In contrast, *RAB6B* (*P*=1.62451E-12), *RAB9B* (*P*=4.4409E-16), and *RAB21* (*P*=9.7542E-12) were significantly down-regulated in primary tumors (*n*=1 097) compared with levels in normal tissue (*n*=114). After integration of prognosis values and mRNA expression levels of these six RAB family genes in BRCA, we concluded that *RAB1B, RAB2A*, and *RAB18* were the most potential biomarkers of BRCA among RAB family genes.

### Correlation among RAB family gene expression levels and BRCA clinicopathological parameters

**Figure 4:**
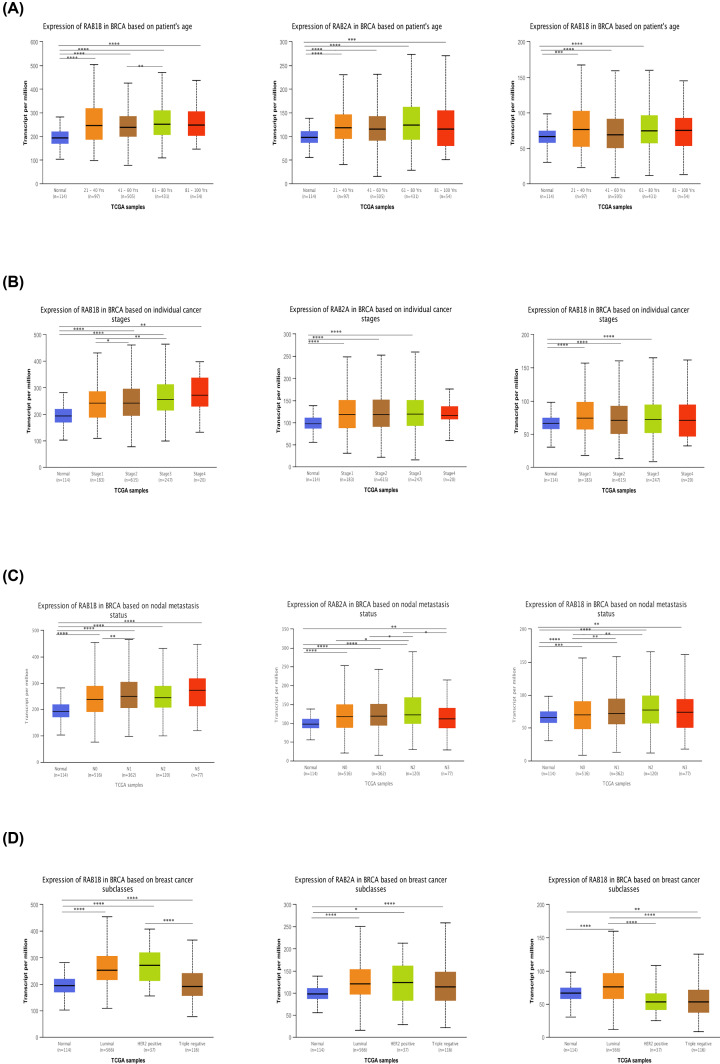
Relationship between *RAB1B, RAB2A* and *RAB18* mRNA expression levels and clinicopathological characteristics in BRCA (**A**) The relationship between mRNA expression levels of these three RAB family genes and patients’ age. (**B**) The relationship between mRNA expression levels of these three RAB genes and individual cancer stages. (**C**) The relationship between mRNA expression levels of these three RAB genes and nodal metastasis status. (**D**) The relationship between mRNA expression levels of these three RAB genes and subclasses. * *P*<0.05; ** *P*<0.01; ***P<0.001; ****P<0.0001.

We further explored the relationships among *RAB1B, RAB2A*, and *RAB18* expression levels and clinicopathological parameters of BRCA patients using UALCAN. As shown in Figure 4A–D, the mRNA transcription levels of the three most potential biomarkers of BRCA were positively correlated with patient age, individual cancer stages, nodal metastasis status and subclasses in BRCA.

### Further exploration of selected RAB genes in BRCA regarding protein expression and functions.

We used the HPA to further explore protein expression and functions of *RAB1B, RAB2A* and *RAB18* in BRCA. As shown in Table 1, these three Rab proteins were mainly located in intracellular, and mainly interacted with ligands closely related to GTP binding and nucleotide binding. They were all got involved in the process of protein transport and transport. Protein expression levels of these three selected genes were further validated higher in BRCA tissues compared that of normal tissues (Supplementary Figure S1).

**Table 1:** Characteristics of *RAB1B, RAB2A* and *RAB18* obtained from the Human Protein Altas database

Gene names	Predicted location	Ligand	Biological process	Gene summary
RAB1B	Intracellular	GTP-binding, Nucleotide-binding	Autophagy, Protein transport, Transport	RAB1B is low molecular mass monomeric GTPase localized on the cytoplasmic surfaces of distinct membrane-bound organelles. RAB1B functions in the early secretory pathway and is essential for vesicle transport between the endoplasmic reticulum (ER) and Golgi.
RAB2A	Intracellular	GTP-binding, Nucleotide-binding	ER-Golgi transport, Protein transport, Transport	The protein encoded by RAB2A belongs to the Rab family, members of which are small molecular weight guanosine triphosphatases (GTPases) that contain highly conserved domains involved in GTP binding and hydrolysis. This protein is a resident of pre-Golgi intermediates, and is required for protein transport from the endoplasmic reticulum (ER) to the Golgi complex. Alternatively spliced transcript variants encoding different isoforms have been found for this gene.
RAB18	Intracellular, Membrane (different isoforms)	GTP-binding, Nucleotide-binding	Protein transport, Transport	RAB18 is a member of a family of Ras-related small GTPases that regulate membrane trafficking in organelles and transport vesicles. Knockdown studies in zebrafish suggest that this protein may have a role in eye and brain development. Mutations in this gene are associated with Warburg micro syndrome type 3. Alternatively spliced transcript variants have been found for this gene.

### Genetic mutations and their association with BRCA prognosis of these three potential biomarkers of BRCA among RAB family genes

**Figure 5:**
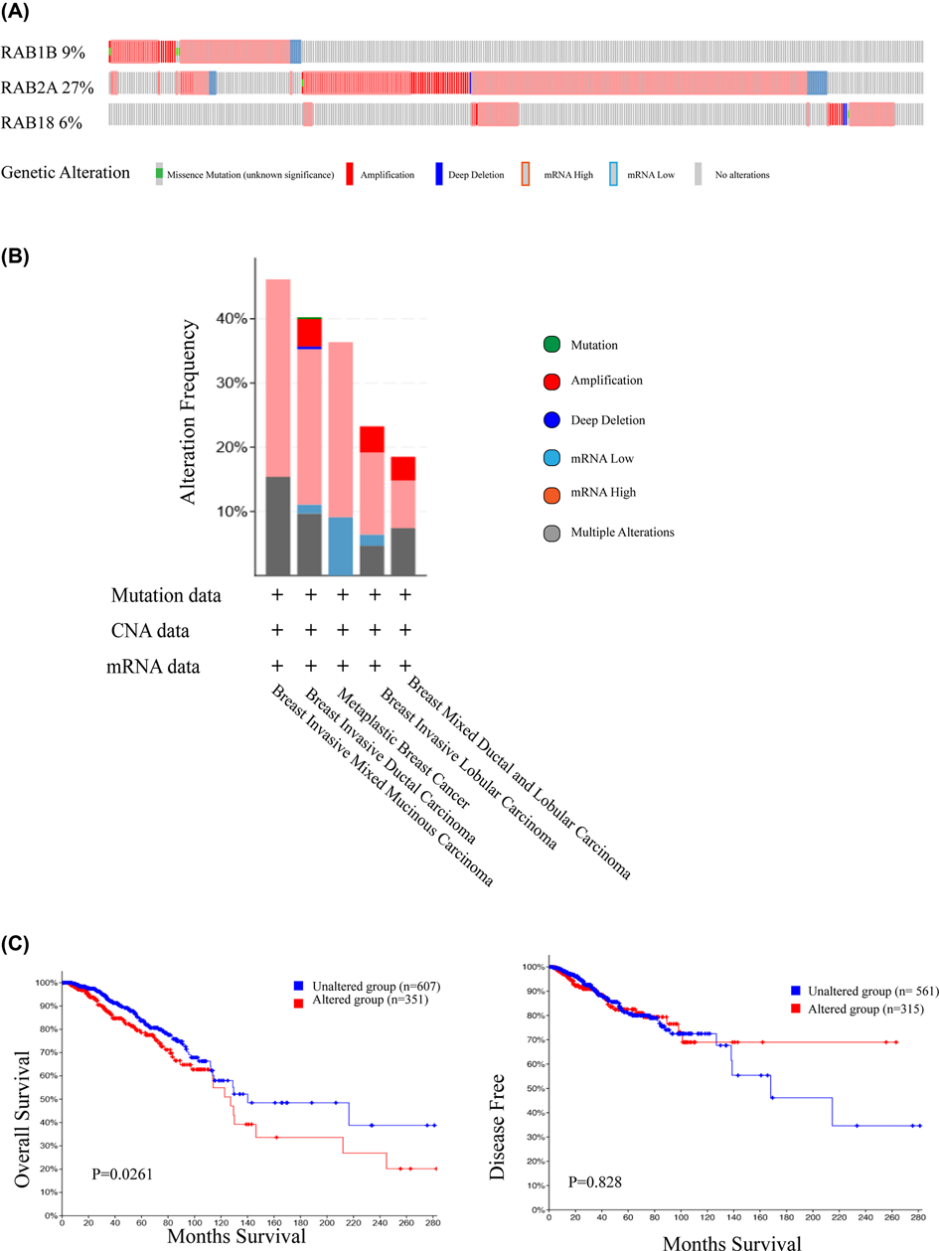
Genetic mutations and their association with BRCA prognosis of *RAB1B, RAB2A* and *RAB18* genes (**A**) OncoPrint of c-BioPortal showed the mutation types and proportions of these three genes, respectively from TCGA samples (**B**) Cancer types summary of c-BioPortal specifically showed the types of mutations and their proportions contained in each cancer type of this selected study (TCGA, Firehose Legacy). (**C**) The genetic mutations of these 3 RAB genes were significantly correlated with patients’ poor OS.

We used c-BioPortal to obtain data from the Cancer Genome Atlas (TCGA) (Firehose Legacy), then explored genetic mutations of *RAB1B, RAB2A*, and *RAB18* in BRCA. As shown in Figure 5A,B, *RAB2A* exhibited the highest mutation rate (27%), followed by *RAB1B* (9%) and *RAB18* (6%). Results showed DNA copy number amplifications and mRNA up-regulation to be the main genetic mutation types. Kaplan–Meier plot curves showed that the genetic mutations were significantly correlated with poor OS in BRCA (*P*=0.0261), although they did not affect DFS (*P*=0.828) (Figure 5C). These findings suggest that the genetic mutations of the three RAB family genes were related to prognosis in BRCA patients.

### Functional enrichment analysis of genes interacting with these three potential biomarkers among RAB family genes

**Figure 6:**
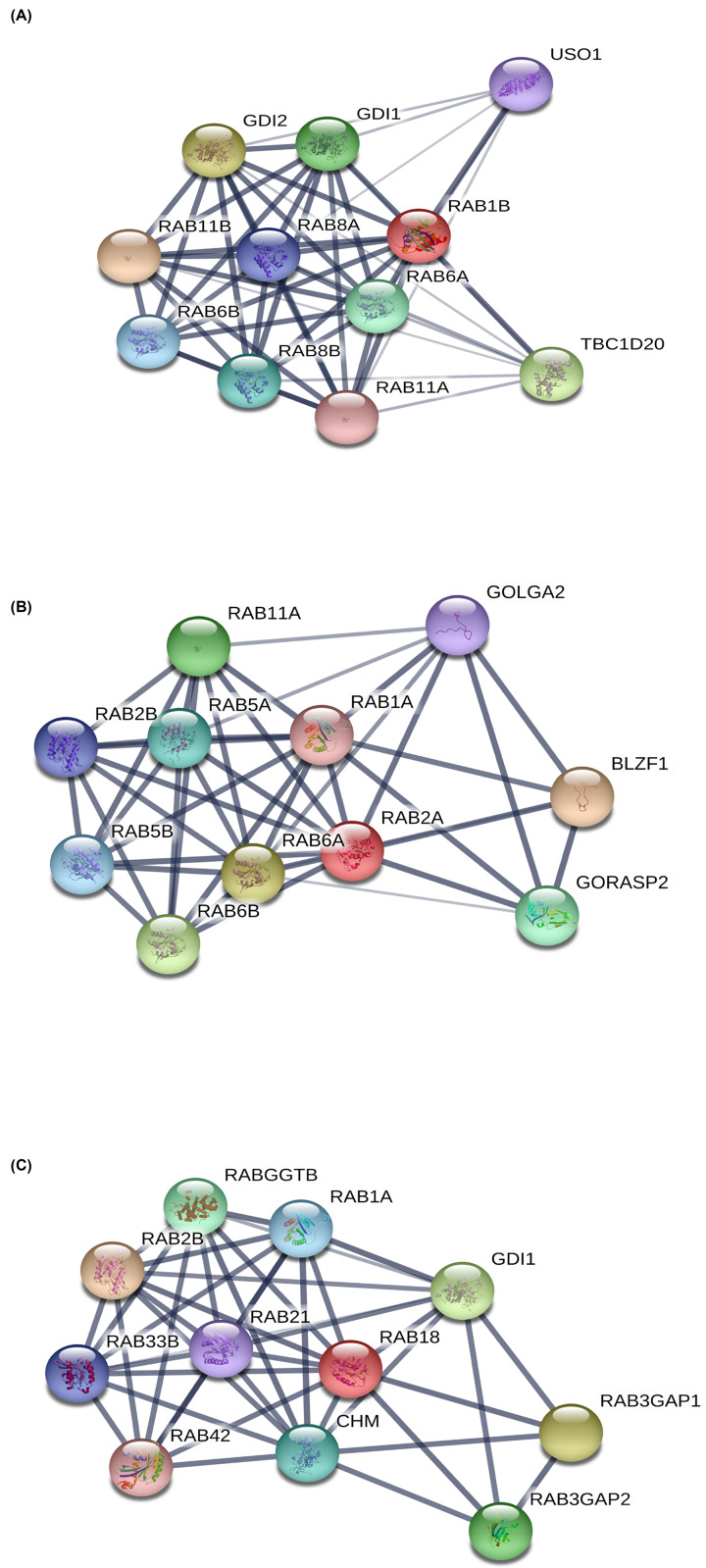
The PPI networks of three RAB family genes using the STRING (**A**) Interaction network of *RAB1B*, (**B**) Interaction network of *RAB2A* (**C**) and Interaction network of *RAB18*.

**Figure 7:**
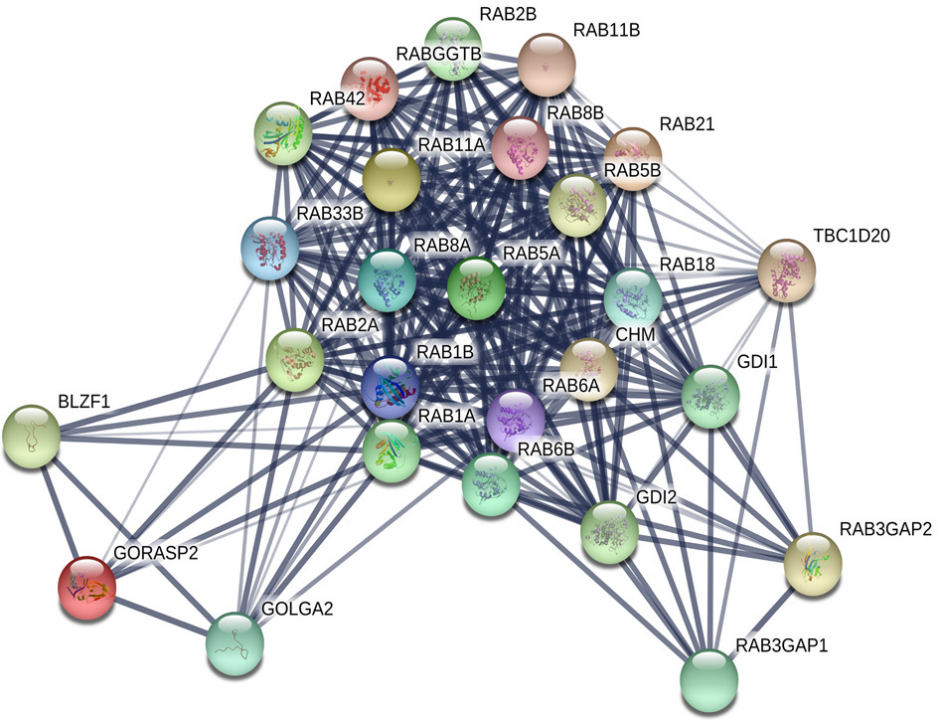
The PPI network of *RAB1B, RAB2A* and *RAB18* and their interacting genes

**Figure 8:**
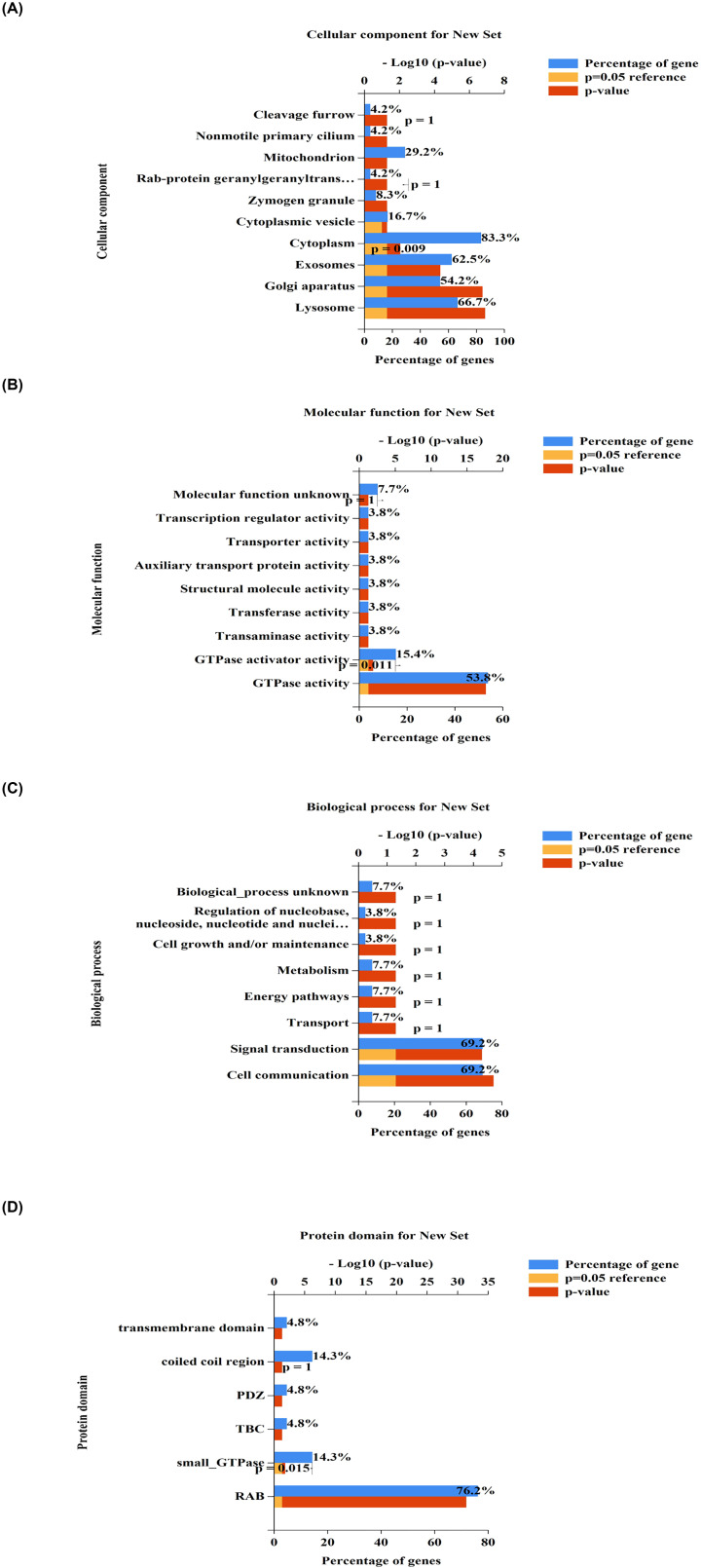
Functional enrichment analysis of three biomarkers and their interacting genes GO functional enrichment analysis and protein domain of interacting genes were performed using Funrich (version 3.1.3). (**A**) cellular components, (**B**) molecular functions, (**C**) biological process and (**D**) protein domain.

To further understand their mechanisms in BRCA, we performed functional enrichment analyses of the three genes (*RAB1B, RAB2A*, and *RAB18*) and their interacting genes. First, we identified the interacting genes corresponding to *RAB1B, RAB2A*, and *RAB18* using STRING. As shown in Figure 6, there were 10, 10, and 10 interacting genes of these three genes, respectively. After removing duplicate genes, a total of 26 genes were further analyzed. As shown in Figure 7, we constructed a protein–protein interaction (PPI) network using STRING. We then imported the 26 genes into FunRich (v3.1.3) for functional enrichment analysis. Results showed that these genes were enriched in cellular components (e.g. exosomes, Golgi apparatus, and lysosomes, Figure 8A); molecular functions (e.g. GTPase activator activity and GTPase activity, Figure 8B); biological processes (e.g. signal transduction and cell communication, Figure 8C); and protein domains (small GTPase and RAB), consistent with our current understanding of Rab proteins (Figure 8D).

## Discussion

In recent years, molecular biology technology and bioinformatics have been widely used in cancer research. This kind of research using public databases containing many samples provides a good way in which to identify tumor biomarkers [20,21]. Here, we systematically explored the roles of RAB family genes in BRCA, including the following characteristics: prognostic values, mRNA expression levels, relationship among expression levels and clinicopathological characteristics, genetic mutations, and gene enrichment. We identified three RAB genes (*RAB1B, RAB2A*, and *RAB18*) as potential biomarkers of BRCA.

Interestingly, these three genes (*RAB1B, RAB2A*, and *RAB18*) have been studied in several malignant tumors. For example, Hiroaki Kajiho et al. found that *RAB2A* is up-regulated in BRCA and causes disease recurrence by controlling E-cadherin polarized Golgi trafficking and MT1-MMP endocytosis [22]. In addition, *RAB18* is reported to be highly expressed in colorectal cancer and closely related to poor survival in patients [23]. The expression of *RAB18* is also significantly increased in hepatocellular carcinoma and significantly associated with poor prognosis due to increased proliferation and metastasis [24]. However, relatively few studies on these genes have been conducted in BRCA. Thus, we hope that our results may encourage research in this field in near future.

We also explored the protein expression levels of *RAB1B, RAB2A*, and *RAB18* in BRCA. However, only the *RAB18* protein was significantly over-expressed (*P*=4.108362E-03) in primary tumors compared with normal tissues in BRCA. The situation was different for *RAB1B* (*P*=8.318090E-01) and *RAB2A* (*P*=8.855370E-02). There may be two reasons for the inconsistency between RNA and protein expression levels. First, the number of patients with protein expression information (normal = 18, primary tumor = 125) was much smaller than the number of patients with mRNA information (normal = 114, primary tumor = 1 097). Second, the process of mRNA translation into protein is regulated by many factors (miRNAs, pseudogenes, circRNAs) at the post-transcription and translation levels, which may result in inconsistent expression between mRNA and protein [25–27].

Human epidermal growth factor receptor 2 (HER2) is a member of the epidermal growth factor receptor (EGFR) family. It is reported that over-expression of HER2 occurs in approximately 20–30% of BRCA patients. Furthermore, up-regulated HER2 is closely related to the poor prognosis of BRCA [28,29]. Previous studies have shown that HER2 does not have a direct activation ligand, and it phosphorylates highly conserved tyrosine residues in the intracellular tyrosine kinase domain mainly through forming a dimer with other EGFR family members (e.g. HER1, HER3) [30,31]. As a result, some key downstream signaling pathways are activated, such as phosphatidylinositol 3 kinase (PI3K) / Akt and mitogen activated protein kinase (MAPK), which have been proved to play important roles in the occurrence and development of BRCA [30–33]. It has been shown that the endocytosis of TFN, EGFR and LDL was related to Rab proteins, and Rab proteins were related to the expression of HER2 [34,35]. Wang et al. revealed that Rab7 could protect HER2 from protease degradation in A431 and MCF7 cell lines [36]. McCaffery et al. also revealed that high expression of RAB4a could increase the internal circulation of HER2 in HELA cell line [37]. In our study, we also found that mRNA of RAB1B and RAB2A were significantly up-regulated in HER2(+) patients compared with normal groups. We hypothesis whether Rab2a and Rab1b also play pivotal roles in BRCA by interacting with HER2, which may provide a new strategy for targeted therapy.

Our study does have some limitations. First, our results were based on public online database information, and therefore remain to be proven by experiments. In the future, we will construct a cell model that differentially expresses the above three genes, and hopefully verify our conclusions both *in vivo* and *in vitro*. Second, although we performed gene enrichment analysis, our study did not elucidate the mechanism by which these three genes participate in BRCA. Therefore, this is one of our future research priorities.

In summary, we have confirmed the over-expression of *RAB1B, RAB2A* and *RAB18* in BRCA, and further validated their prognostic significance in BRCA. We proposed that these differentially expressed genes may be promising molecular targets for the early diagnosis and targeted therapy of BRCA.

## Supplementary Material

Supplementary Figure S1

## Abbreviations

BRCA: breast invasive carcinoma
c-BioPortal: cBio Cancer Genomics Portal
DFS: disease-free survival
GEPIA: Gene Expression Profiling Interactive Analysis
HPA: Human Protein Altas
OS: overall survival
PPI: protein–protein interaction
TCGA: The Cancer Genome Atlas
