# Supplementary material for: Comprehensive analysis of the value of RAB family genes in prognosis of breast invasive carcinoma
Source: Biosci Rep. 2020 May 29;40(5):BSR20201103. doi: 10.1042/BSR20201103 (PMC7260355; doi:10.1042/BSR20201103)
Supplement: Supplementary Figure S1 [file BSR-2020-1103_supp.pdf]

A

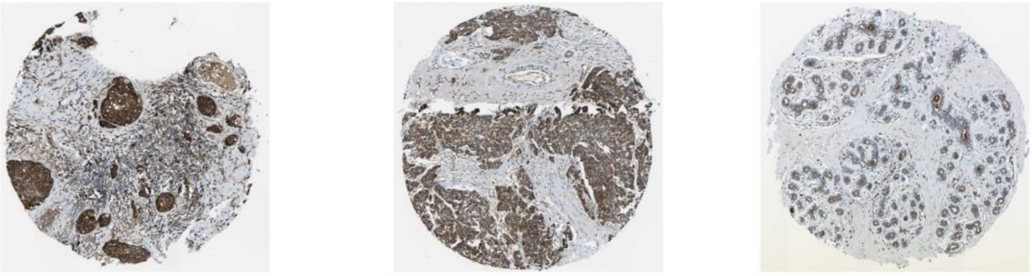

Patient ID: 2392 (Tumor) Patient ID: 2073 (Tumor) Patient ID: 2773 (Normal)

B

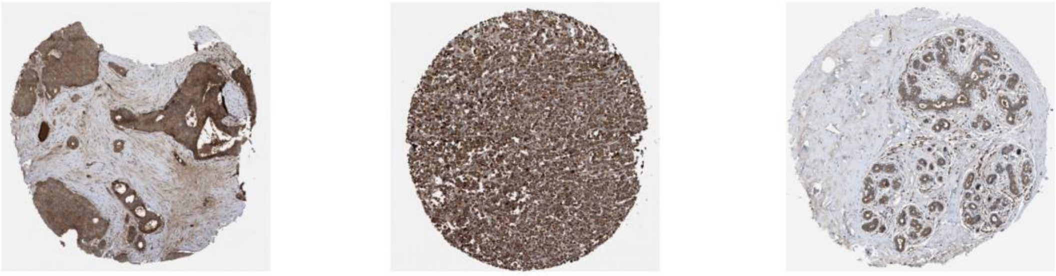

Patient ID: 2428 (Tumor) Patient ID: 1910 (Tumor) Patient ID: 2773 (Normal)

C

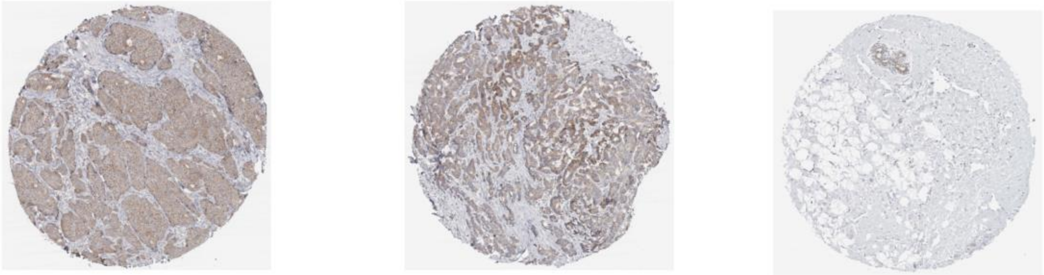

Patient ID: 2428 (Tumor) Patient ID: 1939 (Tumor) Patient ID: 2773 (Normal)

Supplementary Figure.1. Protein expression levels in BRCA tissues and normal breast tissues was obtained using immunohistochemical staining from the HumanProteinAtlas. (A) Protein expression of *RAB1B* (B) Protein expression of *RAB2A* (C) Protein expression of *RAB18*.
